# A Study of the Superplastic Deformation Behavior of Low-Cost Ti-2Fe-0.1B Alloys

**DOI:** 10.3390/ma17061282

**Published:** 2024-03-10

**Authors:** Yaoyao Mi, Yu Lu, Delong Wang, Yihui Zhao, Yuecheng Dong, Hui Chang, I. V. Alexandrov

**Affiliations:** 1 College of Materials Science and Engineering, Tech Institute for Advanced Materials, Nanjing Tech University, Nanjing 211816, China; myy20177030139@163.com (Y.M.); lyu031@163.com (Y.L.); delong426delong426@gmail.com (D.W.); 202221003356@njtech.edu.cn (Y.Z.); ch2006@njtech.edu.cn (H.C.); 2 Department of Materials Science and Physics of Metals, Ufa University of Science and Technology, Ufa 450008, Russia

**Keywords:** titanium alloy, superplastic deformation, globularization, dynamic recrystallization

## Abstract

Titanium alloys have high specific strength and corrosion resistance, which have promising applications in industry. However, the machinability of titanium alloys is limited due to their crystal lattice and physical properties. Thus, in recent years, the superplastic forming of titanium alloys has been intensively developing, in particular, forming at low temperatures and/or high strain rates. In this work, a tensile test of low-cost Ti-2Fe-0.1B alloys was carried out at a temperature of 550~750 °C and a strain rate of 1 × 10^−3^ s^−1^~1 × 10^−2^ s^−1^. The results showed that the alloy exhibited good superplasticity even at a high strain rate (1 × 10^−2^ s^−1^) and a low deformation temperature of 550 °C; the elongation of the alloy in this state reached 137.5%. The high strain rate sensitivity coefficient m (0.3) and the maximum elongation (452%) were obtained at a strain rate of 1 × 10^−3^ s^−1^ and a temperature of 750 °C. Characteristics of the microstructure showed that during superplastic deformation, the recrystallization and grain boundary sliding of the alloy phases were accelerated, which could be ascribed to the effect of the element Fe. At the same time, the TiB phase located around the primary elongated α grains could also induce dynamic recrystallization and dynamic globularization during deformation.

## 1. Introduction

Titanium and titanium alloys are widely used in aerospace, marine, biomedical, and other fields due to their high specific strength, high-temperature resistance, and corrosion resistance [1,2,3]. However, the high deformation resistance and poor machinability of titanium alloys make it difficult to form products using the conventional method, especially products with complex shapes [4]. Thus, superplastic forming (SPF), with the characteristics of low flow stress and uniform plastic deformation at high temperatures, becomes an effective method for manufacturing parts with complex geometric shapes from difficult-to-cut metals [5,6], which could achieve precision shaping and increase material utilization rate [7].

It is well known that superplasticity mainly depends on the grain size, as well as the fraction and distribution of α and β phases [8,9,10]. Because the superplastic deformation mechanism of titanium alloys is mainly grain boundary slip, the finer the grain of titanium alloy, the higher the elongation of the superplastic deformation ability of the alloy [11], so the method for improving superplasticity is mostly related to grain refinement. Severe plastic deformation is a common method for grain refinement, which could lead to excellent superplasticity of titanium alloys. For instance, high elongations in Ti-6Al-4V alloys subjected to high-pressure torsion were observed during tensile tests at relatively low temperatures, 650 °C and 725 °C, even at high strain rates of 10^−2^/s and 10^−1/^s [12]. However, the microstructure with nanocrystalline grains is always unstable and is prone to coarsening at high temperatures [13]. Furthermore, its application in industrial production has been a challenge, given the cost of processing and small-scale production. On the other hand, it is acceptable that the addition of specific alloying elements can not only refine the grain size of alloys but also hinder the migration of grain boundaries and then restrain the growth of crystallization grains during superplastic deformation, thereby maintaining the mechanical properties of the alloy after superplastic forming [14,15]. For instance, A.D. Kotov et al. [15] studied the superplasticity of the Ti-4Al-1V-1Fe-1Ni-0.1B-xMo alloy at 625 °C. The results showed that increasing the Mo content from 1% to 5% increased the m value from 0.38 to 0.50; the flow stress was reduced from 150 MPa to 75 MPa, which is reduced by two times; and the elongation to failure increased from 200% to 700%. The addition of the appropriate β-phase stabilizing element Fe to the titanium alloy can promote the development of diffusion-related deformation mechanisms and effectively reduce the superplastic deformation temperature [16]. Some researchers have found that when the Ti-6Al-4V alloy is added with 2% Fe element, the superplasticity and mechanical properties of the alloy are improved. Fe alloying reduces the superplastic temperature by 100 °C and increases the yield strength by 100 MPa [17,18]. A.D. Kotov et al. [19] studied the effects of different Fe contents (0–2 wt.%) on the microstructure, superplasticity, and mechanical properties of Ti-Al-Mo-V alloys. The results showed that the increase in Fe content promotes recrystallization, fragmentation, and grain boundary slip (GBS) of the phase during superplastic deformation. The alloy with the addition of 0.5% Fe has good superplasticity and mechanical properties at room temperature. On the other hand, it has been reported that the addition of a small amount of B to various pure titanium and titanium alloys can significantly refine the microstructure of the alloy [20,21], which can improve the mechanical properties, toughness, and superplasticity of titanium alloys [22,23]. Qiu et al. [23] found that the TiB phase in the structure of the titanium alloy induces discontinuous dynamic recrystallization via particle-induced nucleation and grain refinement, thereby improving the superplasticity of the alloy. V.A. Kumar et al. [24] found that the addition of the 0.06% B element to the Ti-5Al-5V-5Mo-1Cr-1Fe alloy could refine the grain size, thereby reducing the flow stress of superplastic deformation and improve the elongation to failure of the alloy at high temperature deformation.

Our group designed and developed a low-cost Ti-2Fe-0.1B alloy, which has good mechanical properties, corrosion resistance, and thermal stability [25,26], considering that the alloying elements Fe and B could benefit from the superplasticity of the alloy based on the above mentioned. Therefore, it is interesting to study the high-temperature deformation behavior of the Ti-2Fe-0.1B alloy and explore the mechanism of superplasticity.

## 2. Experimental Methods

### 2.1. Material

The actual chemical composition of the titanium alloy Ti-2Fe-0.1B used in this work is given in Table 1. The Fe element is determined by the Avio-200 ICP-OES/ICP-AES analyzer produced by PerkinElmer Company (Shelton, CT, USA), the ICP-AES analyzer, and the C, O, N, and H elements are determined by the gas analyzer. The Ti-2Fe-0.1B alloy was melted twice in Vacuum Arc Remelting (VAR) melting furnace produced by XinLanHai Automation Technology Co., Ltd. (Shanghai, China) to form an ingot with a diameter of about 420 mm and then forged via the billet opening. The temperature of the first forging ranges from 1020 °C to 1050 °C, the diameter of the specimen is reduced to about 300 mm, the forging is completed in the temperature range of 900 °C to 950 °C, the diameter of the specimen is reduced to 125 mm, and then, via the grinding process, the diameter of the specimen becomes 120 mm. Then, the alloy is rolled from Φ120 mm to the final Φ20 mm bar using a 20-stand continuous rolling process with alternating passes of horizontal and vertical mills under a condition of 836 °C.

### 2.2. High-Temperature Tensile Test

**Figure 1:**
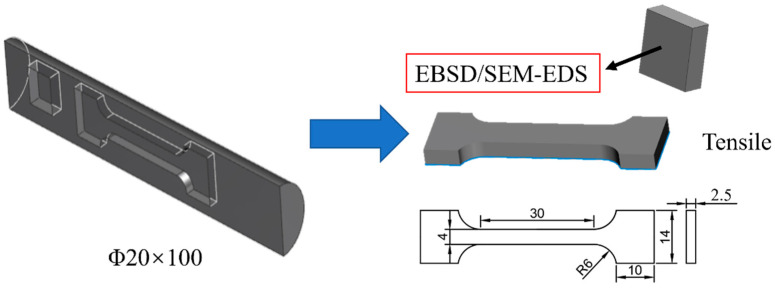
Schematic diagram of experimental samples (mm).

Tensile specimens with a gauge section of 30 mm × 4 mm × 2.5 mm were cut from hot rolled bars along the rolling direction (RD). High-temperature tensile experiments were carried out using an AG-100kNG universal testing machine produced by Shimadzu Company (Kyoto, Japan) at initial strain rates of 1 × 10^−3^/s, 5 × 10^−3^/s, and 1 × 10^−2^/s and temperatures of 550 °C, 650 °C, and 750 °C. Before the experiment, the antioxidant Ti-1 was applied to all test surfaces to minimize or avoid oxidation at high temperatures. During the experiment, the tensile specimens were first heated to the desired temperature and held for 5 min to ensure a fairly uniform temperature distribution throughout the specimen, and then tensile experiments were conducted at a constant strain rate. Three replicate experiments were performed for each state of the specimen. Figure 1 shows the schematic diagram of the experimental samples.

### 2.3. Microstructural Characterization

Electron backscatter diffraction (EBSD), scanning electron microscopy (SEM), and transmission electron microscopy (TEM) were used to characterize the microstructure of the alloys as well as the fracture morphology of the tensile specimens. The samples for EBSD and SEM-EDS observation were cut in the center zone of the bar with the rolling direction, as shown in Figure 1. The X-ray diffraction pattern of the Ti-2Fe-0.1 B alloy illustrates that the Ti-2Fe-0.1B alloy is an α + β two-phase titanium alloy [27]. Samples for EBSD were prepared by electropolishing in 5% perchloric acid and 95% ethanol solution for 120 s at an operating voltage of 40 V. EBSD was carried out using a field emission scanning electron microscope (SEM, JSM-6700F, JEOL, Tokyo, Japan) equipped with an Oxford Instruments EBSD detector and operated at an accelerating voltage of 20 KV. The EBSD scanning step size of all samples was 0.2 μm, and the scanning area was 1600 μm^2^. The data files of the EBSD scans were processed using Channel5.0 data analysis software. The dimensions of the specimens used for SEM microstructure observation were 6 mm × 6 mm × 4 mm. The fracture morphology of the alloys was observed using a JSM-6510 SEM (Tokyo, Japan), and EDS tests were also performed to determine the elemental distribution of the alloys. For TEM studies, the samples were mechanically ground to a thickness of about 100 μm and then jet polished using a Tenupol-5 electrolytic double jet produced by Struers Company (Ballerup, Denmark) in a solution containing 6% perchloric acid, 34% butanol, and 60% methanol, with an accelerating voltage of 25 V and a temperature of −30 °C. Finally, TEM tests were performed on a JEOL JEM-2100 transmission electron microscope with an accelerating voltage of 200 kV.

## 3. Results and Discussion

### 3.1. Initial Microstructure

**Figure 2:**
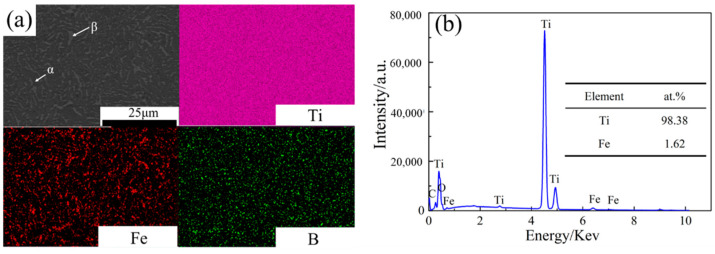
SEM-EDS mapping images of the Ti-2Fe-0.1B alloy. (**a**) phase composition, (**b**) energy spectrum analysis.

The microstructure and element composition of the Ti-2Fe-0.1B alloy were observed and analyzed using SEM-EDS. SEM-EDS mapping of the Ti-2Fe-0.1B alloy and the corresponding energy spectrum analysis are shown in Figure 2. It can be seen that the shape of the β phase is short and rod-like in the Ti-2Fe-0.1B alloy, which is distributed uniformly in α phase substrate. The fraction of the α and β phases was calculated and reached 94.2% and 5.8%, respectively. As a strong eutectoid β stabilizing element for titanium alloy, the Fe element was mainly distributed in the β phase. The content of Fe element by energy spectrum analysis is 1.62%, which is similar to the ICP-AES result. On the other hand, as is well known, the B element does not dissolve neither in the α phase nor in the β phase, which exists in the form of the TiB phase for the titanium alloy. It is obvious that the TiB phase was completely refined in the process of hot forging and continuous hot rolling, which distributes homogeneously in the microstructure.

**Figure 3:**
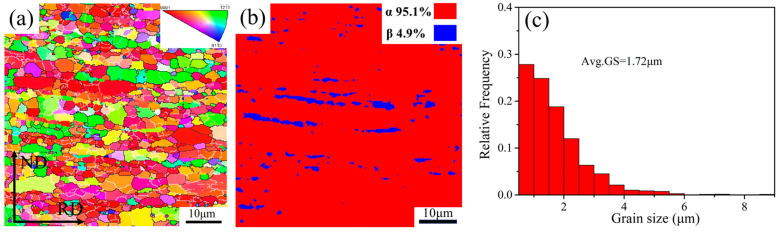
(**a**) IPF image, (**b**) phase composition, and (**c**) average grain size of Ti-2Fe-0.1B alloy.

The inverse pole figure (IPF) and grain size distribution of the Ti-2Fe-0.1B alloy measured by EBSD are shown in Figure 3. Different colors of the IPF plots indicate different orientations of individual grains. Red, green, and blue colors indicate that the test surface of the specimen is parallel to the basal plane {0001}, column plane {112$\bar{0}$}, and conical plane {101$\bar{0}$}, respectively; the remaining colors are transition planes, which also indicate the transitions between orientation differences [28]. The more similar the colors shown in the IPF diagrams, the smaller the orientation difference between individual grains. On the contrary, the orientation difference is larger. It can be seen that the overall microstructure distribution of the Ti-2Fe-0.1B alloy is mainly dominated by the basal plane, and the orientation difference is large. The phase composition of the α and β phase was calculated and reached 95.1% and 4.9%, respectively, which is well corresponding to the SEM results. At the same time, the average grain size is 1.72 μm, which contains a large number of ultrafine grains with a grain size of less than 1 μm and micrometer grains with a grain size of close to 10 μm. It must be noted that the step size of the EBSD test is too small to accurately recognize the grain size below 0.5 μm, traditional hot working was processed in the present work, and the fraction of the grain size in the range of 0–0.5 μm is rare and could be ignored.

### 3.2. Superplastic Deformation

**Figure 4:**
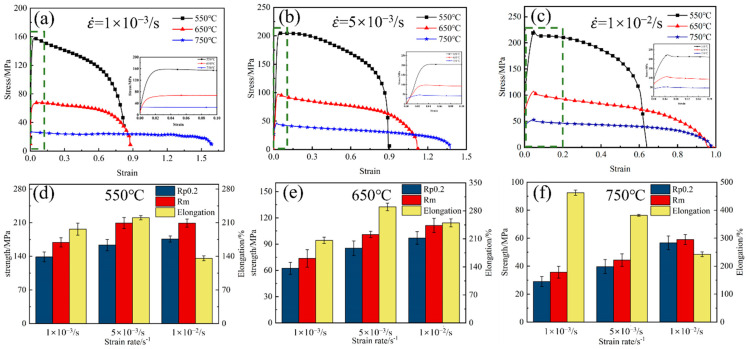
(**a**–**c**) The stress–strain curves and the locally enlarged stress–strain curves of the Ti-2Fe-0.1B alloy under different deformation conditions were obtained. (**d**–**f**) Yield strength, tensile strength, and elongation.

The stress–strain fitting curves of the titanium alloy Ti-2Fe-0.1B at different temperatures and strain rates are shown in Figure 4a–c. All flow curves show similar development, which can be divided into three stages: the elastic deformation stage, strain hardening stage, and flow softening stage. At the initial stage of deformation, the rheological stress increases rapidly as the strain reaches peak stress due to the high work-hardening rate. In the flow-softening stage, the softening rate induced by the dynamic recrystallization (DRX) or dynamic recovery (DRV) mechanism is higher than the hardening rate. When the strain rate is higher or the deformation temperature is lower, the rheological stress decreases significantly with increasing strain. However, when the strain rate is slower or the deformation temperature is higher, the rheological stress decreases slowly with increasing strain and then tends to stabilize.

During superplastic deformation, the rheological stress peak is very sensitive to the deformation parameters. Figure 4d–f show the dynamics of the tensile strength of the material with deformation parameters. Combined with the analysis of Table 2, it is found that the tensile strength decreases with increasing deformation temperature and decreasing strain rate. This is mainly due to the enhancement of thermal activation at high deformation temperatures, the increase in the average kinetic energy of the atoms, and the decrease in the critical shear stress for the occurrence of slip in crystals, which results in a lower impediment to dislocation motion and crystal surface slip. In addition, the cross-slip and climb of dislocations are intensified, resulting in an increase in the recovery rate, which reduces the tensile strength, while high deformation rates lead to work-hardening and strain-rate hardening, which makes the stresses higher [29,30].

**Figure 5:**
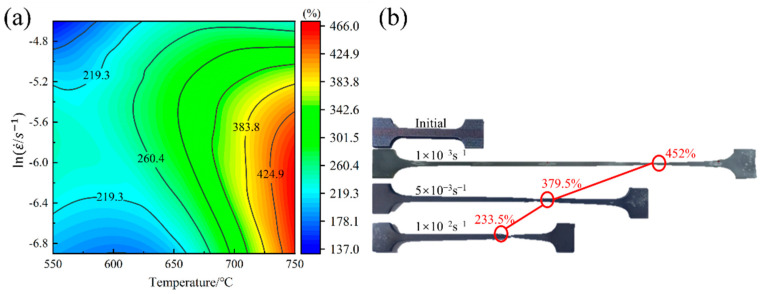
(**a**) Effect of temperature and strain rate on elongation to failure; (**b**) specimens after superplastic tensile deformation at 750 °C and different strain rates.

To further analyze the effect of temperature and strain rate on the elongation of the alloy, a contour map illustrating the elongation distribution is presented in Figure 5a. Combined with Figure 4d–f, it can be seen that a higher deformation temperature and lower strain rate led to greater elongation of the alloy. The minimum elongation achieved was 137.5% at a temperature of 550 °C and a high strain rate (1 × 10^−2^/s). Under other deformation conditions, the maximum elongation exceeded 200%, particularly at 750 °C and a strain rate of 1 × 10^−3^/s, which attained an impressive maximum elongation of 452%. Figure 5b shows the elongation of the Ti-2Fe-0.1B alloy at 750 °C at different strain rates. The sample exhibits an obvious necking phenomenon at a strain rate of 1 × 10^−2^/s. However, at strain rates of 5 × 10^−3^/s and 1 × 10^−3^/s, the sample demonstrates relatively uniform deformation characteristics with minimal necking phenomenon.

A comprehensive analysis shows that with an increasing strain rate, the tensile strength of the alloy increases, and the total elongation decreases. Rheological analysis shows that at the same strain rate, the following best forming properties are achieved in the range of 650–750 °C: low stress, limited hardening, high overall elongation, and no obvious flow softening.

**Figure 6:**
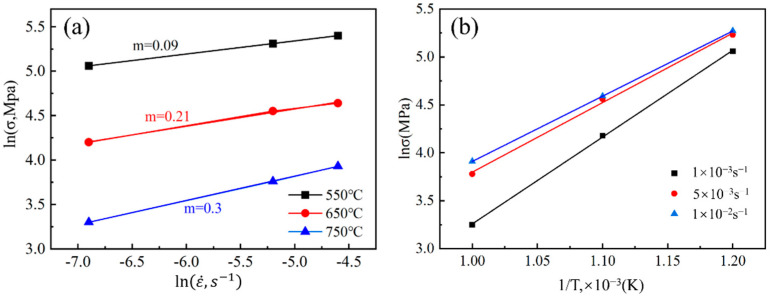
(**a**) Relationship of ln*σ*-ln$\overset{\cdot }{\varepsilon }$ at different temperatures; (**b**) relationship of ln *σ*-(1/*T*) at different strains.

Superplastic deformation of metallic materials is a thermally activated process [31,32,33]. To study the mechanism of material deformation, it is necessary to calculate the values of its deformation activation energy *Q* and strain rate sensitivity factor *m*. The strain rate sensitivity index (*m*) is a critical parameter for characterizing the resistance of superplastic-forming materials to tensile necking. A higher value of *m* indicates that the material is less susceptible to tensile necking and non-uniform strain, thereby enhancing its formability [34]. When *m* ≥ 0.3, the material is generally considered to be a superplastic material [16,35,36], and the larger the value of *m*, the greater the contribution of GBS to superplastic deformation. It is usually defined as the derivative of the true stress–strain rate in the thermostatic true strain double logarithmic plot, denoted as *m* = $\frac{\partial \ln\sigma }{\partial \ln\overset{\cdot }{\varepsilon }}$∣_T,ε_. The ln*σ*-ln$\overset{\cdot }{\varepsilon }$ curves at different deformation temperatures at a strain of 0.03 are shown in Figure 6a. It can be seen that the *m* value is greater than 0.2 when the deformation temperature is 650 °C and 750 °C, and when the deformation temperature is 750 °C, the *m* value of the alloy is 0.3, which indicates that the material has an excellent superplasticity at 750 °C, which is also consistent with the result of material elongation. It can also be concluded that the deformation mechanism of the material is GBS, accompanied by the movement of dislocations [37,38].

In the process of metal forming and deformation, the transition of atoms requires a certain amount of energy. The value of deformation activation energy *Q* can, to a certain extent, reflect the degree of difficulty of deformation of the material and also reflect the degree of difficulty of dislocation activation, recovery, and recrystallization in the process of hot deformation of the material. The relationship between the deformation activation energy *Q* and the rheological stress and deformation parameters during superplastic deformation is generally described using the Arrhenius equation [39,40]:

(1) $$\overset{\cdot }{\varepsilon }=A\cdot \sigma^{n}exp\left(-\frac{Q}{RT}\right)$$

where $\overset{\cdot }{\varepsilon }$, *A*, *σ*, *n*, *Q*, *R*, and *T* represent the strain rate (s^−1^), material constant, flow stress (MPa), stress index (*n* = 1/m), activation energy (kJ/mol), gas constant (8.314 kJ mol^−1^ K^−1^), and absolute temperature (K), respectively. For a fixed strain rate $\overset{\cdot }{\varepsilon }$, the strain activation energy can be expressed by the following equation:

(2) $$Q=R\cdot \frac{\partial \ln\overset{\cdot }{\varepsilon }}{\partial \ln\sigma }\cdot \frac{\partial \ln\sigma }{\partial \left(\frac{1}{T}\right)}=R\cdot \frac{1}{m}\cdot \frac{\partial \ln\sigma }{\partial \left(\frac{1}{T}\right)}$$

Figure 6b shows a plot of the ln*σ*-(1/*T*) relationship (at a fixed strain of 0.03), and the activation energy of superplastic deformation at different strain rates was calculated according to Equation (2). Table 2 shows the strain rate sensitivity factor and deformation activation energy *Q* of the alloy under different deformation conditions. It is generally believed that when the value *Q* of the deformation activation energy of the material approaches the self-diffusion activation energy, the DRV mechanism should be controlled by the dominant dislocation motion during the deformation process. Meanwhile, when the *Q* value is much larger than the self-diffusion activation energy, it indicates that the softening of rheological stresses during the deformation process is caused by dynamic recrystallization [41]. From Table 3, it can be seen that the deformation activation energies of this alloy at temperatures of 550 °C and 650 °C significantly exceed the self-diffusion activation energies of α-titanium (169 kJ/mol) and β-titanium (153 kJ/mol) [42], which implies that dynamic recrystallization is the dominant softening mechanism of alloys with increasing strain in the deformation temperature range of 550–650 °C. The minimum activation energy is 188.26 kJ/mol at 750 °C and 1 × 10^−3^/s, which approaches the self-diffusion activation energy of α-Ti and β-Ti. This indicates that the deformation mechanism of the alloy under this deformation condition is mainly controlled by the dislocation motion.

### 3.3. Microstructure Evolution

**Figure 7:**
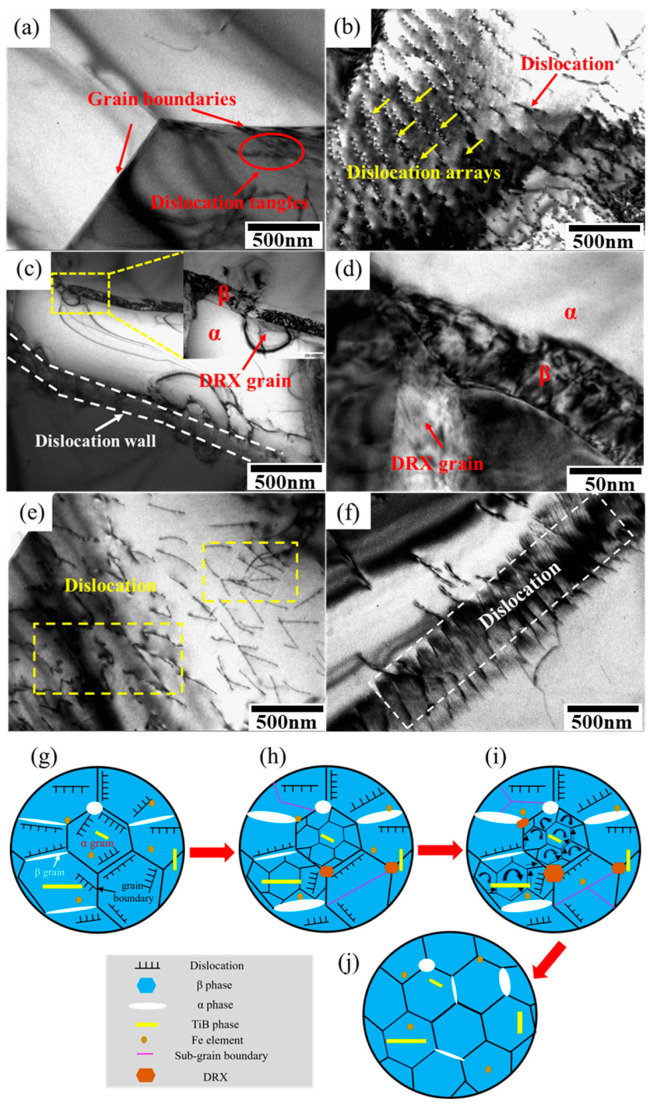
TEM microstructure of the Ti-2Fe-0.1B alloy after superplastic deformation at 750 °C and 1 × 10^−3^/s: (**a**,**b**) dislocation tangles and dislocation arrays; (**c**,**d**) dislocation wall and DRX grains; (**e**) dislocation distribution; (**f**) TiB phase and SAED pattern of TiB phase; (**g**–**j**) microstructure evolution of the alloy during superplastic deformation.

Figure 7 shows the TEM image of the Ti-2Fe-0.1B alloy cut from the near fracture plane after deformation at a temperature of 750 °C and a strain rate of 1 × 10^−3^/s. It has been shown that maintaining a certain amount of intracrystalline dislocations during superplastic deformation is conducive to superplastic deformation because this part of the dislocation motion can provide a certain amount of strain [43]. A large number of dislocation tangles can be observed at the grain boundaries and inside the grains in Figure 7a–f, which indicates that the grain boundary sliding during superplastic deformation is coordinated by the dislocation climbing and sliding around the grain boundaries. Moreover, isometric and parallel dislocation arrays (shown by the yellow arrows) and dislocation walls (indicated by the pink lines) are found in Figure 7b,c, indicating that the dislocation movement inside the grain is very active, and the deformation mechanism of α grains is mainly controlled by the dislocation planar sliding mechanism [44,45]. At the same time, it also shows that strong DRV occurs in the grains [46].

As shown in Figure 7a–c, there are high-density dislocations inside the grain and at the grain boundaries, which promotes dynamic globularization and DRX of the material during superplastic deformation. At the junction of α and β, dislocations are entangled and piled up, which promotes the transformation of the low-angle grain boundary (LAGB) into the high-angle grain boundary (HAGB) and the formation of recrystallized grains (Figure 7c,d), while the formation of recrystallized grains contributes to the grain boundary sliding and grain rotation [37]. In addition, the DRX grains can reduce the flow stress and significantly improve the plasticity during high-temperature deformation [47].

It is well known that the B element does not dissolve neither in the α phase nor in the β phase, which only exists in the titanium alloy in the form of the TiB phase. On the other hand, it is acceptable that the existence of the TiB phase could hinder the dislocation sliding and then lead to the dislocation accumulation. Accompanied by dislocation proliferation around the TiB phase, as well as high-temperature deformation, the nucleation of DRX occurred, and the formation of new grains started, which could adapt to superplastic deformations during high temperatures [48,49]. Besides that, as studied by Maria N. Postnikova [50], the 0.01–0.1% B decreased the flow stress values at the initial stage of superplastic deformation and provided a stable flow due to the facilitation of recrystallization and globularization effects.

To more clearly demonstrate the evolution of the microstructure and the mechanism of superplastic deformation of the Ti-2Fe-0.1B alloy, Figure 7g–j show the microstructure evolution during the superplastic deformation of the Ti-2Fe-0.1B alloy. Initially, the microstructure of the as-received alloy is composed of high-density dislocations caused by hot rolling, which were mainly distributed inside the α grains, α/β grain boundaries, and around the TiB phase (Figure 7g). As a result of the tensile test, with increasing strain and deformation temperatures, the occurrence of DRV causes the formation of sub-grains and the development of a structure as more dislocations accumulate at the sub-grain boundaries. This causes the transformation of LAGB to HAGB and the generation of DRX grains. The fraction of the β-phase increased as a result of the occurrence of DRX and phase transitions, as well as an increase in the sizes of primary α and β grains due to the static growth and a reduction in dislocations due to static recovery (Figure 7h). Sub-grains around TiB formed new grains due to the DRX. At the same time, a large number of dislocations slip and aggregate in the α grains to form sub-grains, which recrystallize and form new α grains with an increase in strain. Adjacent grain boundaries switch along the shear plane; in addition, GBS also promotes spheroidization while rotating along the grains (Figure 7i). Finally, the DRX grains grow with increasing deformation time (Figure 7j). Additionally, the Fe element also promotes recrystallization, spheroidization, and grain boundary sliding of the phase during superplastic deformation [19,51].

### 3.4. Fracture Morphology

**Figure 8:**
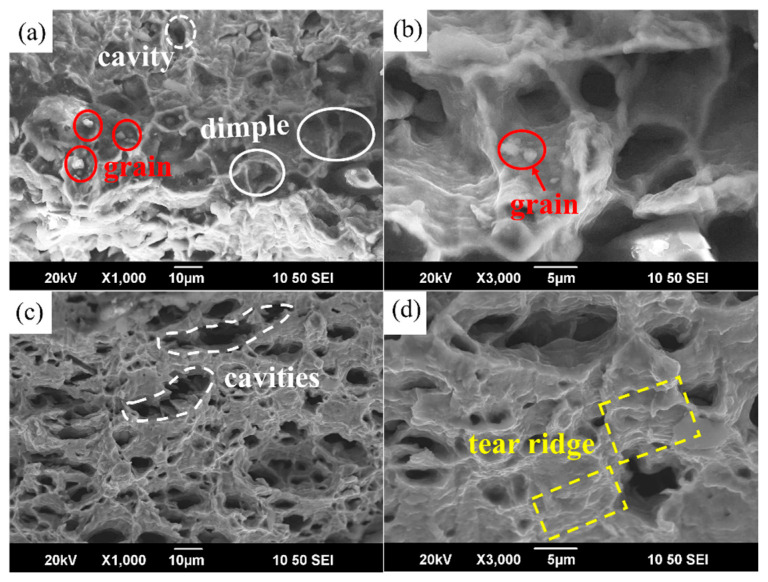
Fracture morphology (**a**–**d**) of the Ti-2Fe-0.1B alloy after superplastic deformation: (**a**,**b**) 1 × 10^−3^/s and 750 °C and (**c**,**d**) 1 × 10^−2^/s and 550 °C, respectively.

Figure 8 shows the fracture morphology of the Ti-2Fe-0.1B alloy after superplastic deformation. It can be seen from the figure that before the tensile fracture, the sample has a large plastic deformation, and the fracture morphology of the specimen under different deformation conditions contains fracture dimples with a distribution of cavities. In Figure 8a,b, the dimples are large and densely distributed, and there are small grains in the cavity. It seems that the cavity first nucleates on these small particles and then aggregates, grows, and joins under the combined action of GBS and plastic deformation so that a number of cracks are formed on the surface of the specimen. The cracks connect to each other; then, this leads to the fracture of the sample. In Figure 8c,d, there are dimples with plastic fracture characteristics and a large number of tear ridges, which are typical ductile fracture characteristics. The dimples contained in the micromorphology are elongated due to the shear stress of the specimen before fracture.

In this paper, the superplastic deformation mechanism and the microstructure evolution of the Ti-2Fe-0.1B alloy are investigated. The following conclusions can be made:

(1) The average grain size of the Ti-2Fe-0.1B alloy reached 1.72 μm due to the effect of Fe and B elements, which gave the alloy good superplasticity even at a high strain rate (1 × 10^−2^ s^−1^) and a low deformation temperature of 550 °C. The elongation of the alloy reached 137.5% under this deformation condition. The high strain rate sensitivity coefficient m (0.3) and the maximum elongation (452%) were obtained at a strain rate of 1 × 10^−3^ s^−1^ and a temperature of 750 °C.
(2) The TiB phase provides nucleation sites for DRX and induces significant DRX and dynamic globularization during superplastic deformation. The main mechanism of superplastic deformation of the Ti-2Fe-0.1B alloy is grain boundary sliding under the coordination of dislocation motion, accompanied by DRX.

By analyzing the fracture morphology, it is found that the fracture of the alloy contains dimples of plastic fracture under high temperature and low strain rate tensile deformation. The aggregation, growth, and connection of cavities cause cracks to appear on the surface of the sample, which leads to its destruction. 

## Figures and Tables

**Table 1:** Chemical composition of Ti-2Fe-0.1B alloy (wt.%).

Fe	B	C	O	N	H	Ti
1.89	0.08	0.014	0.062	0.004	0.0012	Balance

**Table 2:** Yield strength, tensile strength, and elongation of Ti-2Fe-0.1B alloy under different deformation conditions.

	Strain Rate/s^−1^	Rp0.2/MPa	Rm/MPa	Elongation/%
550 °C	1 × 10^−3^/s	138.7 ± 10.3	169.0 ± 9.6	196.5 ± 12.6
	5 × 10^−3^/s	163.3 ± 11.7	209.3 ± 11.4	220.3 ± 4.5
	1 × 10^−2^/s	176.0 ± 6.6	209.0 ± 8.5	135.8 ± 5.2
650 °C	1 × 10^−3^/s	62.3 ± 6.8	73.7 ± 10.0	206.3 ± 7.7
	5 × 10^−3^/s	85.3 ± 8.4	101.0 ± 3.6	289.8 ± 9.5
	1 × 10^−2^/s	97.0 ± 7.2	111.3 ± 8.1	250.0 ± 10.0
750 °C	1 × 10^−3^/s	29.0 ± 3.6	35.7 ± 4.2	462.3 ± 9.3
	5 × 10^−3^/s	39.7 ± 5.0	44.3 ± 4.5	381.5 ± 3.0
	1 × 10^−2^/s	56.7 ± 4.9	59.0 ± 3.6	242.5 ± 8.3

**Table 3:** Values of strain rate sensitivity factor m and thermal activation energy Q under different deformation conditions.

Deformation Conditions		*m*	Q (kJ/mol)
Strain Rate (s^−1^)	Temperature (°C)		
1 × 10^−3^–1 × 10^−2^	550	0.09	835.94
	650	0.21	286.91
	750	0.3	188.26
Average value		0.2	437.04

## Data Availability

Data are contained within the article.
